# Protective role of IL-17-producing γδ T cells in a laser-induced choroidal neovascularization mouse model

**DOI:** 10.1186/s12974-023-02952-1

**Published:** 2023-11-25

**Authors:** Yu-Hsien Chang, Chung-Hsi Hsing, Chiao-Juno Chiu, Yi-Rou Wu, Sheng-Min Hsu, Yu-Hsiang Hsu

**Affiliations:** 1 Institute of Clinical Medicine, College of Medicine, National Cheng Kung University, Tainan, Taiwan; 2 Department of Anesthesiology, Chi Mei Medical Center, Tainan, Taiwan; 3 Department of Medical Research, Chi Mei Medical Center, Tainan, Taiwan; 4 Department of Ophthalmology, National Cheng Kung University Hospital, College of Medicine, National Cheng Kung University, Tainan, Taiwan; 5 Research Center of Clinical Medicine, National Cheng Kung University Hospital, College of Medicine, National Cheng Kung University, Tainan, Taiwan; 6 Antibody New Drug Research Center, National Cheng Kung University, Tainan, Taiwan

**Keywords:** AMD, IL-17A, Inflammation, Choroidal neovascularization, Oxidative stress

## Abstract

**Background:**

Vision loss in patients with wet/exudative age-related macular degeneration (AMD) is associated with choroidal neovascularization (CNV), and AMD is the leading cause of irreversible vision impairment in older adults. Interleukin-17A (IL-17A) is a component of the microenvironment associated with some autoimmune diseases. Previous studies have indicated that wet AMD patients have elevated serum IL-17A levels. However, the effect of IL-17A on AMD progression needs to be better understood. We aimed to investigate the role of IL-17A in a laser-induced CNV mouse model.

**Methods:**

We established a laser-induced CNV mouse model in wild-type (WT) and IL-17A-deficient mice and then evaluated the disease severity of these mice by using fluorescence angiography. We performed enzyme-linked immunosorbent assay (ELISA) and fluorescence-activated cell sorting (FACS) to analyze the levels of IL-17A and to investigate the immune cell populations in the eyes of WT and IL-17A-deficient mice. We used ARPE-19 cells to clarify the effect of IL-17A under oxidative stress.

**Results:**

In the laser-induced CNV model, the CNV lesions were larger in IL-17A-deficient mice than in WT mice. The numbers of γδ T cells, CD3^+^CD4^+^RORγt^+^ T cells, Treg cells, and neutrophils were decreased and the number of macrophages was increased in the eyes of IL-17A-deficient mice compared with WT mice. In WT mice, IL-17A-producing γδ T-cell numbers increased in a time-dependent manner from day 7 to 28 after laser injury. IL-6 levels increased and IL-10, IL-24, IL-17F, and GM-CSF levels decreased in the eyes of IL-17A-deficient mice after laser injury. In vitro*,* IL-17A inhibited apoptosis and induced the expression of the antioxidant protein HO-1 in ARPE-19 cells under oxidative stress conditions. IL-17A facilitated the repair of oxidative stress-induced barrier dysfunction in ARPE-19 cells.

**Conclusions:**

Our findings provide new insight into the protective effect of IL-17A in a laser-induced CNV model and reveal a novel regulatory role of IL-17A-producing γδ T cells in the ocular microenvironment in wet AMD.

**Supplementary Information:**

The online version contains supplementary material available at 10.1186/s12974-023-02952-1.

## Background

Age-related macular degeneration (AMD) is a retinal disease that causes damage to the macula and leads to loss of central vision. AMD is usually frequent in elderly people, and there are two types of macular degeneration associated with age: dry (atrophic) and wet (exudative) AMD [1]. Most AMD cases start as the dry type, and approximately 10–20% of individuals with dry AMD tend to progress to the wet type [2, 3]. AMD occurs in both eyes but does not necessarily progress at the same pace in both eyes. Wet AMD is associated with choroidal neovascularization (CNV), which is characterized by the proliferation of blood vessels behind the retina that begin to grow toward the macula and tend to leak fluid into the macula, causing macular damage that leads to severe central vision loss [4]. The reduction in tissue oxygenation causes the production of vascular endothelial growth factor (VEGF), a critical factor involved in the development of wet AMD [5].

The clinical efficacy of intravitreal anti-VEGF drugs has been widely demonstrated in wet AMD [6]. However, long-term anti-VEGF therapy for some patients with wet AMD may have a poor outcome [7, 8]. Under physiological conditions, VEGF can provide critical trophic support necessary for retinal function [9]. In addition, VEGFA inhibition might lead to retinal pigment epithelium (RPE) and photoreceptor cell death and vision loss [10]. An improved understanding of CNV pathogenesis is still crucial for the prevention and treatment of AMD.

Reactive oxygen species (ROS) formation is a byproduct of the normal metabolism of oxygen and plays important roles in cell signaling and homeostasis [11]. ROS levels dramatically increase, and ROS accumulate during a process called oxidative stress, which may damage cell structures [11]. Recent studies have investigated the role of excess ROS in the pathogenesis and development of AMD [12, 13]. The antioxidant capacity of retinal cells decreases with age, increasing oxidative stress, which causes irreversible damage to photoreceptors and RPE cells [14]. The upregulation of Nrf2 activity and the expression of HO-1 can suppress retinal cell apoptosis [15]. Therefore, improving the endogenous defense system against ROS damage in the retina is important.

Cytokines are critical mediators of communication for the immune system. TNF-α stimulates the expression of proangiogenic VEGF [16] and facilitates pathologic angiogenesis in AMD [17]. IL-6 is significantly associated with the volume of macular edema in patients with CNV [18]. IL-10 regulates macrophage function and promotes pathological angiogenesis [19]. Therefore, cytokine dysregulation is believed to play a critical role in the pathogenesis of AMD.

IL-17A is a pleiotropic cytokine and is mainly produced by T helper 17 cells (Th17 cells), type three innate lymphoid cells (ILC3s), and gamma delta T cells (γδ T cells). IL-17A binds to a heterodimeric receptor complex, which consists of IL-17RA and IL-17RC, to activate the MAPK and NF-κB signaling pathways and further induce the production of chemokines. These chemokines further recruit immune cells, such as monocytes and neutrophils, to sites of local inflammation. Therefore, IL-17A is critical for host defense against microbes because it promotes the recruitment of neutrophils and induces the production of antimicrobial peptides [20–22]. IL-17-driven inflammation is normally controlled by regulatory T (Treg) cells and the anti-inflammatory cytokines IL-10, TGF-β, and IL-35 [23].

There is much evidence indicating that AMD is associated with the immune system, which includes complement, macrophages, and T cells [24, 25]. Th17 cells are the main source of IL-17A and are involved in the pathogenesis of autoimmune diseases, including multiple sclerosis [26, 27], systemic lupus erythematosus [28], and inflammatory bowel disease [29, 30]. Treg and Th17 cells are two described lymphocyte subsets with opposing actions. Th17 cells represent a proinflammatory subset, whereas Treg cells have an anti-inflammatory effect. The Th17/Treg balance plays a critical role in the development of autoimmune diseases [31, 32]. In addition, γδ T cells are an early cellular source of IL-17A, which promotes neutrophil recruitment [33].

Previous studies reported that serum IL-17A levels were elevated in patients with AMD [34, 35]. However, the functional roles of IL-17A in AMD progression remain incompletely understood. In this study, we aimed to investigate the role of IL-17A in CNV development and verify the effects of IL-17A in a laser-induced CNV mouse model.

## Materials and methods

### Animals

Wild-type (WT) C57BL/6J mice were purchased from the National Laboratory Animal Center (Taipei, Taiwan). IL-17A-deficient mice on a C57BL/6J genetic background were maintained in the animal center of National Cheng Kung University. Both IL-17A-deficient and WT mice (6–8 weeks old) were used and kept on a 12-h light–dark cycle at 22 ± 2 °C. The research procedures were approved by the Animal Ethics Committee of National Cheng Kung University (IACUC Approval no. 111266). The methods were carried out in accordance with the approved guidelines.

### Laser-induced CNV mouse model and fluorescence angiography

Mice were anesthetized by intraperitoneal injection of Zoletil (8 mg/kg; VIRBAC, Carros, France) and Rompun (0.32 mg/kg; Bayer, Leverkusen, Germany), and the pupils were dilated with one drop of 1% tropicamide ophthalmic solution (Santen Pharmaceutical Co., Ltd, Kita-ku, Osaka, Japan). After anesthesia and dilation, CNV was induced with a 532-nm laser photocoagulator (250 mW, 50 μm spot size, duration 80 ms, four spots per eye) with a microscopic delivery system (Micron IV, Phoenix Research Laboratories, Pleasanton, CA, USA). In all eyes, four spots were applied around the optic disc and between retinal vessels four times. A bubble formed immediately at the site of laser application, indicating successful rupture of Bruch’s membrane. Spots with subretinal hemorrhage following laser photocoagulation were excluded. Fundus fluorescein angiography (FFA) was used to evaluate CNV leakage by performing with the retinal imaging microscopy (Micron IV, Phoenix Research Laboratories) 7, 14, 21, and 28 days after laser photocoagulation. Mice were anesthetized, and their pupils were dilated and intraperitoneally injected with 2% sodium fluorescein (Sigma‒Aldrich, St. Louis, MO, USA) at 20 μg/gram body weight. The FFA images were taken with a retinal imaging microscope at 5 min after fluorescein injection. The fluorescein leakage area of each lesion was contoured and evaluated quantitatively with ImageJ software (National Institutes of Health, Bethesda, MD, USA).

### Isolation of ocular-infiltrating cells

To isolate the immune cells in the retina and choroid, the tip of angled scissors was inserted between the skin and the eyeball, and then the mouse eyes were enucleated. To avoid contamination, the eye was briefly dipped in 70% ethanol and washed in PBS. The anterior segment (cornea, iris, and lens) was removed. Both eyes were pooled for each animal and enzymatically digested in DMEM-F12 serum-free medium (GeneDireX, Taiwan) supplemented with 2 mg/ml dispase II (Sigma‒Aldrich) at 37 °C for 40 min. The posterior segment of the eyes, including the sclera, choroid, and retina, was then disrupted in the digestion medium. The sample was filtered with a 70 μm cell strainer (CORNING, Tewksbury, MA, USA) and then centrifuged at 4 °C (1800 rpm/610 g for 5 min). The supernatant was removed, and the cells were resuspended in medium for further flow cytometry analysis.

### Fluorescence-activated cell sorting (FACS) analysis and detection of cytokine-expressing lymphocytes

The cells were suspended in FACS buffer (7.7 mM NaN3, 2 mM EDTA and 2% FBS dissolved in PBS) to produce a single-cell suspension. Myeloid cells were stained with surface marker antibodies, including BV 421-conjugated anti-CD45 (BD Pharmingen™, San Diego, CA, USA), 7-AAD-conjugated anti-CD11b (BD Pharmingen™), Alexa 647-conjugated anti-F4/80 (eBioscience, San Diego, CA, USA), Alexa 488-conjugated anti-Ly6G (BD Pharmingen™), and Alexa 647-conjugated anti-Ly6C (BioLegend, San Diego, CA, USA). Lymphoid cells were stained with surface marker antibodies including PE-conjugated anti-CD45 (BD Pharmingen™), BV 421-conjugated anti-TCRγδ (BD Pharmingen™), and Alexa 488-conjugated anti-CD4 (BioLegend), in the dark for 30 min at 4 °C. For transcription factor staining, cells were fixed and permeabilized for 30 min at 4 °C using BD Cytofix/Cytoperm buffer (BD Biosciences, La Jolla, CA, USA), washed twice with BD Perm/Wash buffer and then stained with transcription factor marker antibodies, including 7-AAD-conjugated RORγt (BD Pharmingen™) and Alexa 647-conjugated Foxp3 (BioLegend), in the dark for 30 min at 4 °C. For intracellular cytokine staining, cells isolated from the retina were activated with 20 ng/ml PMA (Sigma‒Aldrich), 1 μM ionomycin (Sigma‒Aldrich), and BFA (1:1000, BD Pharmingen™) and cultured for 5 h in 5% CO_2_ at 37 °C. Cells were stained with surface marker antibodies including APC-Cy7-conjugated anti-CD45 (BD Pharmingen™) and PE-conjugated anti-CD4 (BD Pharmingen™). Then, the cells were fixed and permeabilized for 30 min at 4 °C using BD Cytofix/Cytoperm buffer (BD Biosciences), washed twice with BD Perm/Wash buffer and then stained with Alexa 488-conjugated anti-IL-17A (BD Pharmingen™) in the dark for 30 min at 4 °C. The cell samples were acquired using a BD FACS Canto II Flow Cytometer (BD Biosciences) using BDFACS DIVA software (BD Biosciences) and analyzed with FlowJo software (v.10, FlowJo™).

### Histological analysis and immunofluorescence staining

Eyes from mice were harvested and fixed in neutral buffered 4% paraformaldehyde. After paraffin embedding, retinal cross-sections were cut perpendicularly (5 μm). Hematoxylin and eosin (H&E) staining and immunofluorescence staining were performed following standard protocols. For immunofluorescence staining, the primary antibodies used were anti-IL-17A (diluted to 2 μg/ml; Genetex, Alton Pkwy Irvine, CA, USA) and anti-ZO-1 (1:50 dilution; Abcam, Cambridge, UK). Isotype-matched control antibodies were used as a negative control. The samples were washed with PBS and stained with fluorescence-conjugated secondary antibody at room temperature for 1 h. The slides were mounted with ProLong^®^ Gold antifade reagent with DAPI (Invitrogen, Waltham, MA, USA). Images were taken using a scanning confocal laser microscope (Olympus FV1000, Waltham, MA, USA) to visualize the stained cells.

### Cytokine analysis

IL-17A concentrations in serum from mice with laser-induced CNV and healthy mice were measured using a mouse IL-17A ELISA Kit (R&D Systems, Minneapolis, MN, USA) according to the manufacturer’s instructions. To analyze dynamic changes in cytokine levels in healthy mice and mice with laser-induced CNV on day 7, day 14, day 21, and day 28, serum samples were collected and evaluated using a customized LEGENDplex™ kit according to the manufacturer’s instructions (BioLegend). The bead-based multiplex assay panel allowed simultaneous quantification of IL-17F, IL-17A, IL-22, IL-23, TNF-α, IFN-γ, IL-5, IL-13, and IL-6 using a sandwich immunoassay. Data were acquired on a BD FACSCanto II (BD Biosciences). The data were analyzed using BioLegend’s LEGENDplex™ data analysis software.

### Real-time quantitative polymerase chain reaction (RT‒qPCR)

Total RNA was extracted from total mouse ocular cells or cultured cells with QIAzol Lysis Reagent (QIAGEN, Hilden, Germany) according to the manufacturer’s instructions. RNA samples (1 μg) were then subsequently used for reverse transcription using the PrimeScript™ RT Reagent Kit (Perfect Real Time) (Takara, Japan), and the cDNA samples were further diluted 10 times in diethylpyrocarbonate (DEPC)-treated water. cDNA was then amplified on a thermocycler (Roter-Gene Q detection system; QIAGEN) with gene-specific primers. The PCR mixture contained 2 µl of diluted cDNA samples, 5 µl of SYBR Green Master Mix (QIAGEN), 1.6 μl of RNase-free water (QIAGEN), and 0.7 µl of each primer at 10 pmol/µl in a final reaction volume of 10 μl. The amplification conditions were as follows: activation at 95 °C for 2 min, followed by 40 cycles of denaturation at 95 °C for 10 sec and extension at 60 °C for 20 sec. mRNA quantification analysis results were normalized to β-actin or GAPDH, which was used as the internal control. Relative fold changes in mRNA expression were determined by calculating 2^–ΔΔCt^. All sequences of primers used in this study are listed in Additional file 1: Table S1.

### Cell culture

The ARPE-19 human retinal pigment epithelial cell line was obtained from the American Type Culture Collection (ATCC). ARPE-19 cells were cultured in a 1:1 mixture of DMEM/Ham’s F-12 medium with L-glutamine and 15 mM HEPES (GeneDireX) and were supplemented with 2.48 g/L sodium bicarbonate (Avantor, Radnor, PA, USA), 10% FBS (HyClone™, Logan, UT, USA), and 1% antibiotic–antimycotic (GeneDireX). Cells were incubated at 37 °C with 95% air and 5% CO_2_.

### Cell viability assay

ARPE-19 cells were cultured in 96-well plates and incubated in DMEM/F12 containing IL-17A (10–100 ng/ml; GenScript, Piscataway, NJ, USA) or 300 μM H_2_O_2_ (Honeywell Fluka™, Seelze, Germany) for 24 h. After treatment, the cells were incubated with 3-(4,5-dimethylthiazol-2-yl)-2,5-diphenyltetrazolium bromide (MTT; VWR, Radnor, PA, USA) at a final concentration of 0.2 mg/ml for 3 h. The MTT solution was removed, and 150 μl of DMSO (Sigma‒Aldrich) was added to each well. The optical densities at 570 nm were read on a microplate spectrophotometer.

### Apoptosis assay

ARPE-19 cells were grown overnight in a 12-well plate at a density of 8 × 10^4^ cells/well and then treated with 300 μM H_2_O_2_ (Honeywell Fluka™) and IL-17A (100 ng/ml; GenScript) in DMEM/F12 with 2% FBS for 24 h. Cells were harvested and stained with a FITC Annexin V apoptosis detection kit (BD Pharmingen™ 556,547) according to the manufacturer’s instructions. The cells were subjected to apoptosis analysis using a BD FACSCalibur (BD Biosciences).

### Permeability assay

ARPE-19 cells were seeded on 24-mm diameter, 0.4-μm pore size, polyester Transwell filters (Falcon, Tewksbury, MA, USA) at a density of 1 × 10^4^ cells/well overnight. Cells were cultured in DMEM/F12 with 1% FBS for 7 days until they reached the appropriate cell confluence. The cells were then treated with 300 μM H_2_O_2_ and IL-17A (100 ng/ml) for 16 h. After treatment, 10 kDa FITC-dextran (100 μg/ml; Sigma–Aldrich) was added to the upper chamber of the Transwell, and then collected 50 μl aliquots from the lower chamber at 10–16 h to detect and measure the fluorescence signal at 485/538 nm excitation/emission wavelengths using a luminometer.

### Western blotting

Mouse ocular tissues or ARPE-19 cells were lysed with commercial Cell Lysis Buffer (#9803, Cell Signaling, Beverly, MA, USA). Proteins were separated by SDS‒PAGE and transferred electrophoretically to 0.45 μm PVDF membranes (Millipore, Burlington, MA, USA). The membranes were blocked with 5% (w/v) nonfat milk in TBST for 1 h at room temperature and then incubated overnight at 4 °C with primary antibodies: anti-NQO-1 (1:5000; Cell Signaling), anti-HO-1 (1:10,000; Cell Signaling), anti-Nrf2 (1:500; Santa Cruz, Dallas, TX, USA), anti-GAPDH (1:5000; Genetex) and anti-tubulin (1:5000; Abcam). After binding of primary antibodies, the membranes were washed four times with TBST and incubated for 1 h at room temperature with species-specific horseradish peroxidase-labeled secondary antibodies. After binding of secondary antibodies, the membranes were washed five times with TBST. The binding of secondary antibodies was detected with SuperSignal West Pico Chemiluminescent Substrate (Millipore), and the chemiluminescence signals were visualized and imaged on a luminescence imaging system following exposure and development of Hyperfilm ECL molecules (Invitrogen).

### Statistical analysis

Prism 9.0 (GraphPad Software; San Diego, CA, USA) was used for the statistical analysis. GraphPad Prism software was employed to process the initial data and plot graphs. Normality tests were performed before statistical tests. In this study, the data were normally distributed, and variances were homogeneous in all groups; parametric analysis (t test for the comparison of two groups or one-way ANOVA with Tukey's multiple comparisons test for the comparison of three or more groups) was used, and data are presented as the mean ± SEM. Two-way ANOVA with Sidak's multiple comparisons test was used to analyze multiple groups. The specific test that was used is indicated in the figure legends. P < 0.05 was considered to indicate statistical significance.

## Results

### IL-17A deficiency exacerbated the severity of laser-induced CNV in a mouse model

**Fig. 1:**
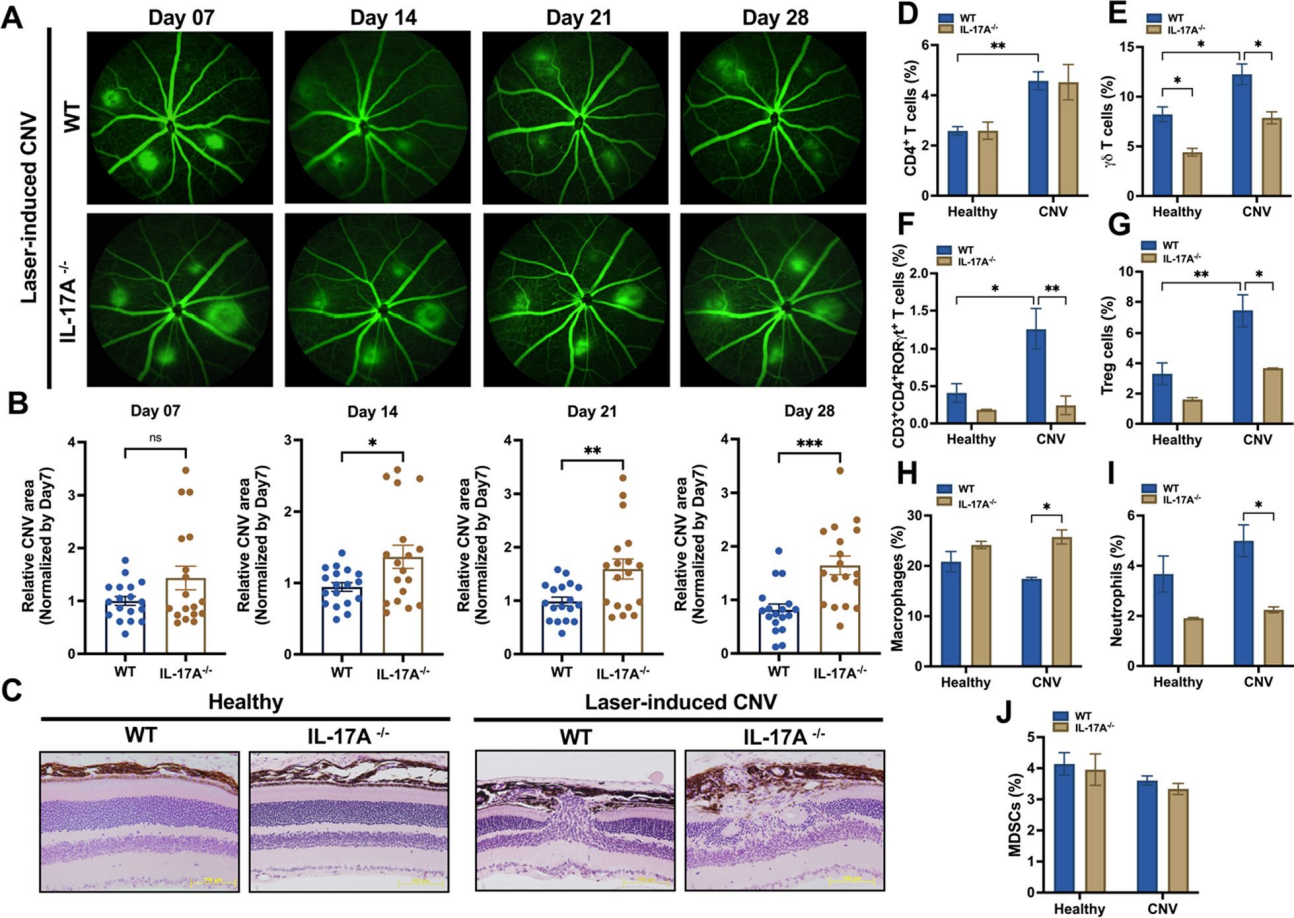
IL-17A deficiency exacerbated disease severity in a laser-induced CNV mouse model. **A** Representative fundus fluorescein angiography (FFA) images from WT and IL-17A^−/−^ mice at 7, 14, 21, and 28 days after laser photocoagulation. CNV lesions were analyzed by fluorescence angiography. **B** Quantification of the CNV area after laser photocoagulation at 7, 14, 21, and 28 days. Statistical differences were determined by unpaired *t* test. Data are presented as the mean ± SEM (*P < 0.05, ** < 0.01, *** < 0.001). N = 18 mice per group. **C** H&E staining of the retinal structure in healthy mice and WT and IL-17A^−/−^ mice with laser-induced CNV at 28 days after laser injury. Magnification: 200X. Scale bar = 100 μm. **D**–**J** Representative flow cytometry data for the CD4^+^ T cell, γδ T cell, CD3^+^CD4^+^RORγt^+^ T cell, Treg cell, macrophage, neutrophil, and MDSC populations in the eyes of WT and IL-17A^−/−^ mice 28 days after laser injury. N = 5 mice per group. Statistical differences were determined by two-way ANOVA and Sidak’s multiple comparisons test. Data are presented as the mean ± SEM (*P < 0.05, **P < 0.01)

A previous study [35] reported that AMD patients had higher IL-22 and IL-17 levels than healthy individuals. IL-17 indirectly enhances vascular endothelial cell growth [36]. However, the precise role of IL-17A in AMD in vivo is still incompletely understood. To clarify the effect of IL-17A on neovascular AMD, we established a laser-induced CNV mouse model in WT and IL-17A-deficient mice and used fluorescein to observe the severity of CNV. Based on available knowledge, we hypothesized that the severity of CNV would decrease in IL-17A-deficient mice. However, we unexpectedly observed that the lesions in laser-induced CNV in IL-17A-deficient mice were more extensive than those in WT mice (Fig. 1A). Quantitative image analysis showed that there was no significant change between groups on day 7; however, the CNV area was significantly increased in IL-17A-deficient mice compared with WT mice at 14, 21, and 28 days after laser injury (Fig. 1B). H&E staining showed that the damage to retinal structure was more severe in IL-17A-deficient mice than in WT mice 28 days after laser injury (Fig. 1C). We also observed that the protein level of VEGFA was not significantly different in WT and IL-17A-deficient mice in the laser-induced CNV mouse model 28 days after laser injury; however, VEGFA mRNA was upregulated in WT mice compared with IL-17A-deficient mice 28 days after laser injury (Additional file 1: Fig. S1A–D).

### IL-17A deficiency influenced ocular Treg cells, CD3^+^CD4^+^RORγt^+^ T cells, γδ T cells, macrophages, and neutrophils in a laser-induced CNV mouse model

Ocular immunity is involved in both maintaining visual homeostasis and driving AMD pathogenesis. We investigated whether distinct immune cell populations infiltrate the retina in WT and IL-17A-deficient mice after laser injury. We collected cervical lymph nodes from WT and IL-17A-deficient mice at 28 days after laser injury to analyze CD3^+^CD4^+^RORγt^+^ T cells, Treg cells (CD3^+^CD4^+^Foxp3^+^), and γδ T cells (CD3^+^TCRγδ^+^). FACS analysis showed no significant differences in these immune cell populations in the cervical lymph nodes of WT and IL-17A-deficient mice after laser injury (Additional file 1: Fig. S2A–C). At the same time, we also collected retina and choroidal tissue to analyze immune cell populations in the eyes (Additional file 1: Fig. S3). FACS analysis showed that the percentages of ocular CD3^+^CD4^+^RORγt^+^ T cells, Treg cells, and γδ T cells were lower in IL-17A-deficient mice than in WT mice at 28 days after laser injury (Fig. 1D–G). We further investigated whether IL-17A plays a role in recruiting the infiltration of innate immune cells (macrophages, MDSCs, and neutrophils) into the eyes and subsequently affects the extent of choroidal neovascularization. We collected retina and choroidal tissue to analyze the cell populations of macrophages (CD11b^+^F4/80^+^), MDSCs (CD11b^+^Ly6G^−^Ly6C^hi^), and neutrophils (CD11b^+^Ly6G^+^Ly6C^+^) in WT and IL-17A-deficient mice after laser injury. FACS analysis showed that IL-17A deficiency reduced neutrophil recruitment but increased macrophage infiltration into the eyes at 28 days after laser injury (Fig. 1H–J).

### Th17 cells and γδ T cells were the main cellular sources of ocular IL-17A in mice with laser-induced CNV

**Fig. 2:**
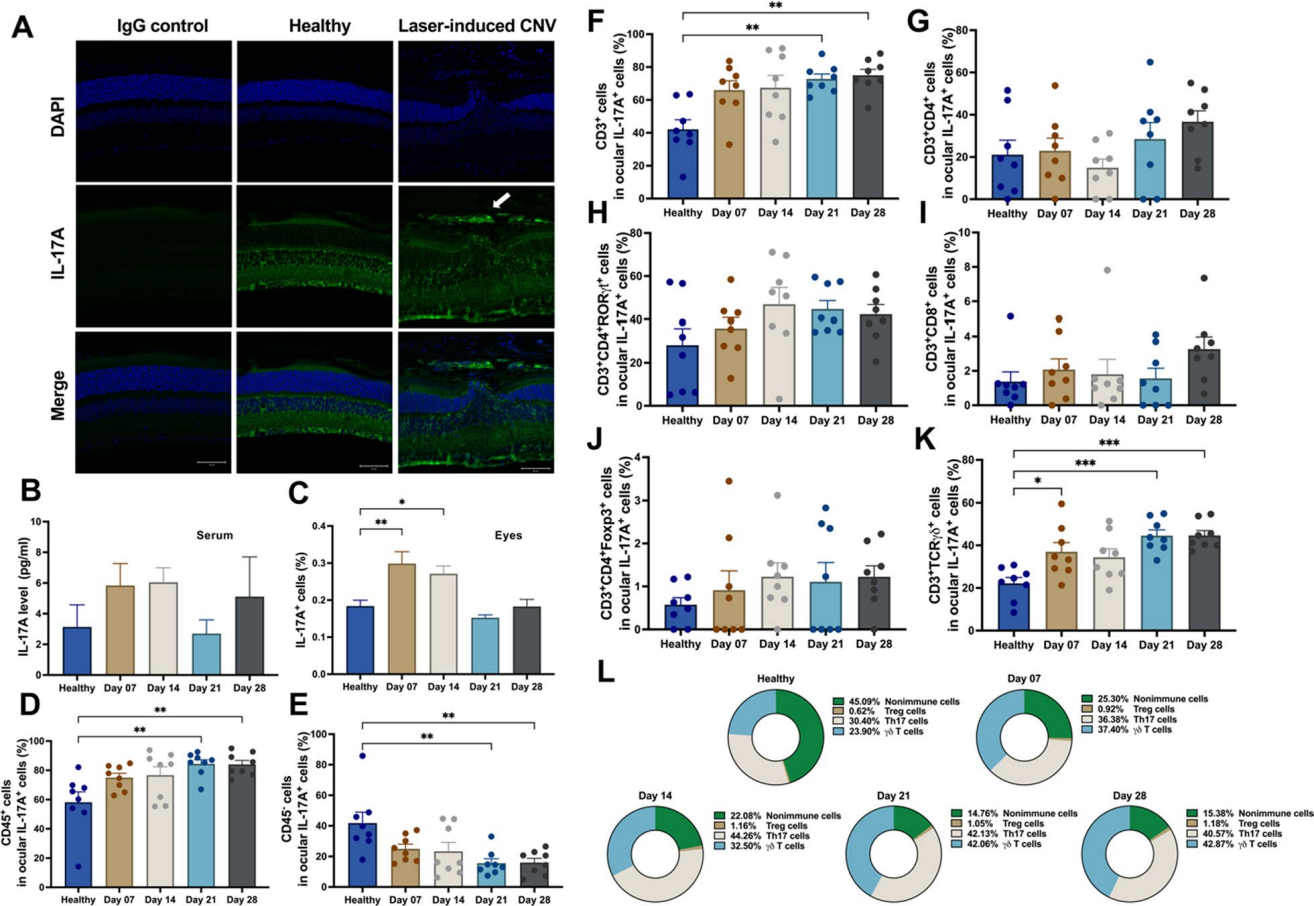
Cellular source of IL-17A in WT mice with the laser-induced CNV. **A** Immunofluorescence staining of ocular tissue with an anti-IL-17A antibody in vivo. Rabbit IgG isotype was used as the negative control. Magnification: 200X. Scale bar = 50 μm. **B** Serum IL-17A levels were measured by using ELISA at 7, 14, 21 and 28 days after laser photocoagulation. N = 5 mice per group. **C**–**E** Flow cytometry analysis of IL-17A-expressing cells among ocular cells and further gating of immune cells (CD45^+^) and nonimmune cells (CD45^−^). Statistical differences were determined by one-way ANOVA and Tukey’s multiple comparisons test. Data are presented as the mean ± SEM (*P < 0.05, **P < 0.01). N = 8 mice per group. **F**–**K** Representative flow cytometry data for the CD3^+^IL-17A^+^ T cell, CD3^+^CD4^+^IL-17A^+^ T cell, CD3^+^CD8^+^IL-17A^+^ T cell, γδ T cell, Th17 cell, and Treg cell populations in the eyes of WT mice at 7, 14, 21, and 28 days after laser injury in comparison with healthy control mice. N = 8 mice per group. Statistical differences were determined by one-way ANOVA and Tukey’s multiple comparisons test. Data are presented as the mean ± SEM (*P < 0.05, **P < 0.01, ***P < 0.001). The data shown are representative of three independent experiments with similar results. **L** The dynamic changes in the proportion of IL-17A-expressing immune cells and the percentages of nonimmune cells, Treg cells, Th17 cells, and γδ T cells were analyzed at 7, 14, 21, and 28 days after laser injury. N = 8 mice per group

To investigate the involvement of IL-17A in the laser-induced CNV mouse model, we performed immunofluorescence staining to detect the localization of IL-17A in healthy mice and mice with laser-induced CNV. IL-17A was expressed in the inner retinal layer in both healthy mice and mice with laser-induced CNV. Interestingly, IL-17A was also expressed in laser-induced CNV lesions in mice (Fig. 2A). ELISA showed that the serum level of IL-17A was not significantly different between healthy mice and mice with laser-induced CNV (Fig. 2B), which suggested that IL-17A may play an important regulatory role in local laser-induced eye lesions in mice. FACS analysis showed that the number of IL-17A-expressing cells in the mouse retina was increased after laser injury (Fig. 2C). To verify the possible cellular source of IL-17A in the laser-induced CNV mouse model, we collected retina and choroidal tissue from healthy mice and mice with laser-induced CNV on days 7, 14, 21, and 28 (Additional file 1: Fig. S4). FACS analysis showed that the percentage of CD45^+^IL-17A^+^ immune cells increased and that of CD45^−^IL-17A^+^ nonimmune cells decreased from day 7 to 28 after laser injury (Fig. 2D, E). We found that Th17 cells (CD3^+^CD4^+^RORγt^+^IL-17A^+^) and γδ T cells (CD3^+^TCRγδ^+^IL-17A^+^) were the two major cellular sources of IL-17A in the eyes of mice with laser-induced CNV (Fig. 2F–K). In addition, we analyzed the dynamic changes in the proportion of IL-17A-expressing immune cells and found that the percentage of γδ T cells increased in a time-dependent manner from day 7 to 28 after laser injury (Fig. 2L).

### IL-17A deficiency influenced the expression of ocular proinflammatory molecules in a laser-induced CNV mouse model

**Fig. 3:**
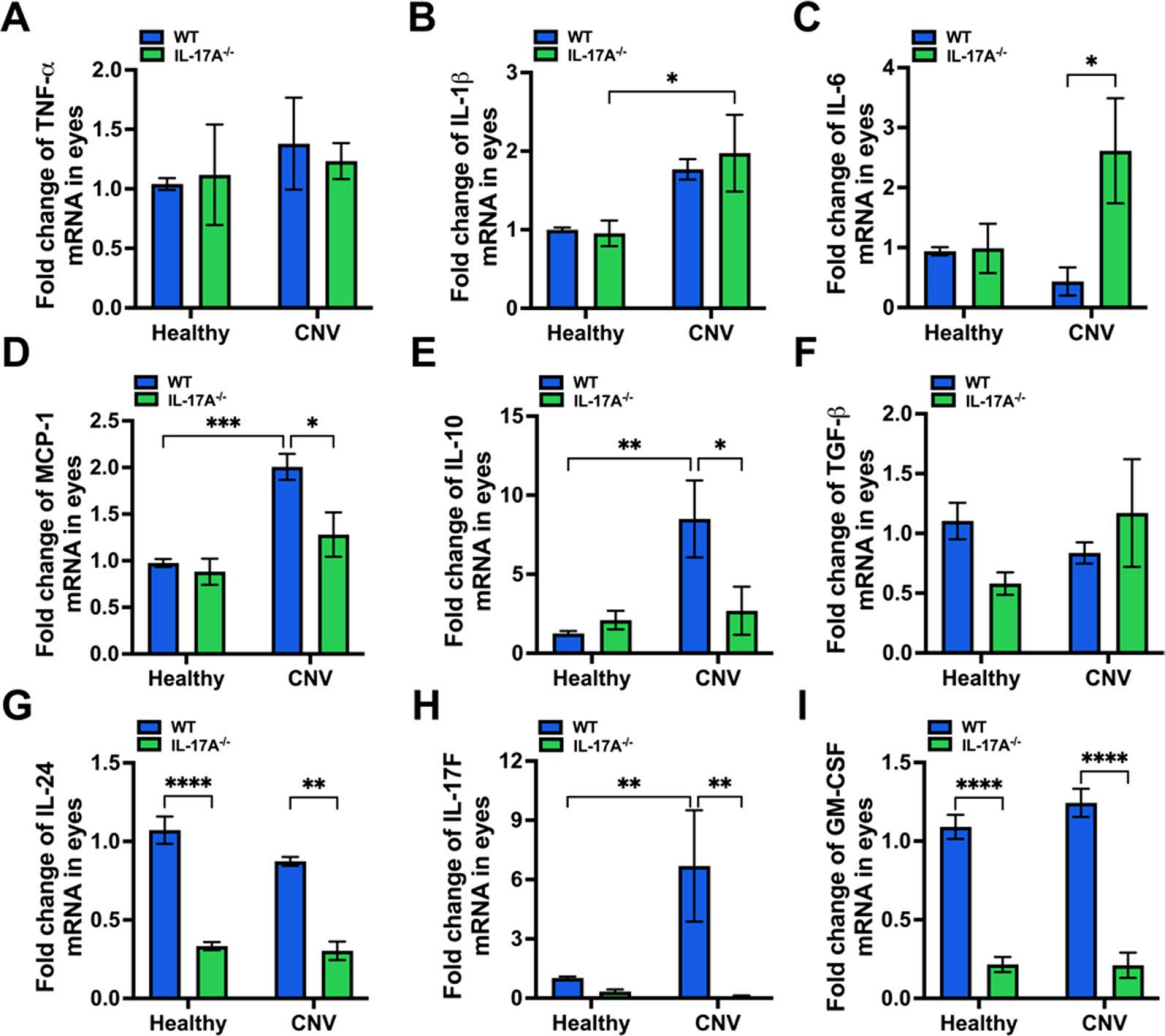
IL-17A deficiency influenced ocular cytokine expression in a laser-induced CNV mouse model. Eyes were harvested from healthy mice and WT and IL-17A^−/−^ mice with laser-induced CNV at 28 days after laser injury (N = 8). Both eyes were pooled for each animal. **A**–**I** The mRNA expression of IL-10, MCP-1, IL-1β, TNF-α, IL-6, TGF-β, IL-24, IL-17F, and GM-CSF in the eyes was analyzed by using RT‒qPCR with specific primers. Statistical differences were determined by two-way ANOVA and Sidak’s multiple comparisons test. Data are presented as the mean ± SEM (*P < 0.05, **P < 0.01, ***P < 0.001, ****P < 0.0001). The data shown are representative of three independent experiments with similar results

We further analyzed the inflammatory response in the eyes after laser injury. We performed RT‒qPCR to investigate the expression of proinflammatory cytokines (TNF-α, IL-1β, IL-6, and MCP-1), anti-inflammatory cytokines (IL-10 and TGF-β), and Th17-associated cytokines (IL-17F, GM-CSF, and IL-24). There was no significant change in TNF-α, IL-1β, and TGF-β expression between IL-17A-deficient and WT mice (Fig. 3A, B and F). IL-17A deficiency increased IL-6 and decreased MCP-1 and IL-10 expression in the eyes after laser injury (Fig. 3C–E). IL-17F, GM-CSF, and IL-24 levels were significantly reduced in IL-17A-deficient mice compared with WT mice at 28 days after laser injury (Fig. 3G–I). In addition, we performed a cytometric bead assay to further measure dynamic changes in systemic cytokine levels (Th1-, Th2-, and Th17-associated cytokines) in a laser-induced CNV mouse model. We observed an increasing trend for IFN-γ and IL-6 levels and a decreasing trend for IL-22 levels in IL-17A-deficient mice compared with WT mice (Additional file 1: Fig. S5A–I).

### IL-17A inhibited H_2_O_2_-induced apoptosis in ARPE-19 cells

**Fig. 4:**
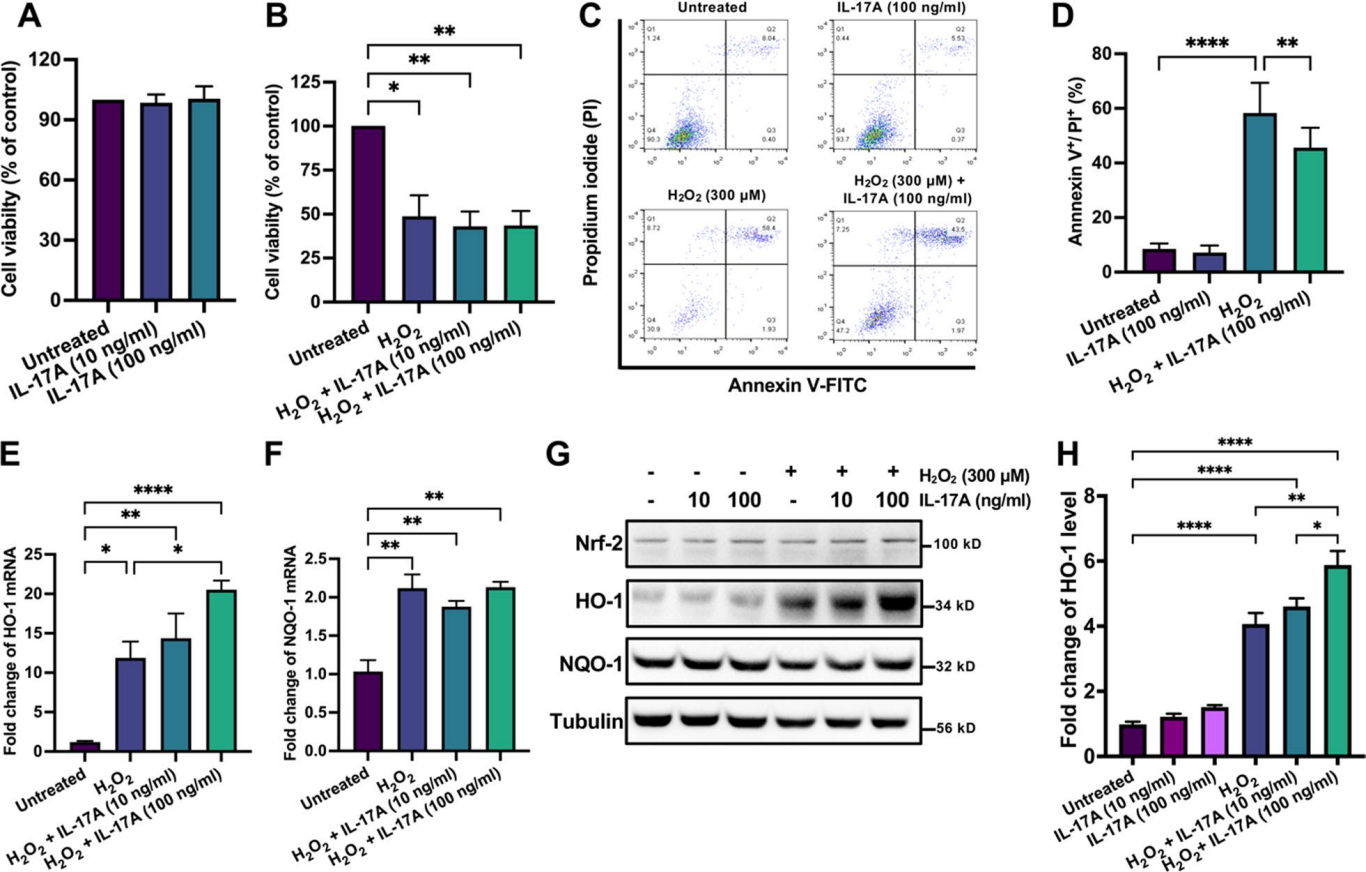
IL-17A inhibited H_2_O_2_-induced apoptosis through HO-1 in ARPE-19 cells. **A**, **B** ARPE-19 cells were treated with IL-17A (10-100 ng/ml), H_2_O_2_ (300 μM), and IL-17A (100 ng/ml) plus H_2_O_2_ (300 μM) for 24 h. Cell viability was analyzed using the MTT assay. Statistical differences were determined by one-way ANOVA and Tukey’s multiple comparisons test. Data are presented as the mean ± SEM (*P < 0.05, **P < 0.01). **C** ARPE-19 cells were treated with or without IL-17A (100 ng/ml) for 24 h and then treated with H_2_O_2_ (300 μM) for 24 h, and cell apoptosis was analyzed using FACS. **D** Quantification of Annexin V^+^/ PI^+^ ratios in ARPE-19 cells after H_2_O_2_ and IL-17A treatment. Statistical differences were determined by one-way ANOVA and Tukey’s multiple comparisons test. Data are presented as the mean ± SEM (**P < 0.01, ****P < 0.0001). The data shown are representative of three independent experiments with similar results. **E**, **F** ARPE-19 cells were cotreated with H_2_O_2_ and IL-17A for 8 h, and the mRNA levels of HO-1 and NQO-1 were analyzed by RT‒qPCR with specific primers. N = 3. Statistical differences were determined by one-way ANOVA and Tukey’s multiple comparisons test. Data are presented as the mean ± SEM (*P < 0.05, **P < 0.01, ****P < 0.0001). The data shown are representative of three independent experiments with similar results. **G**, **H** ARPE-19 cells were cotreated with H_2_O_2_ and IL-17A for 24 h. The cell lysates were collected and analyzed using immunoblotting with specific antibodies against Nrf2, HO-1, and NQO-1. Tubulin was used as an internal control. Statistical differences were determined by one-way ANOVA and Tukey’s multiple comparisons test. Data are presented as the mean ± SEM of three independent experiments. (*P < 0.05, **P < 0.01, ****P < 0.0001)

Previous studies [12, 13, 37, 38] have indicated that oxidative stress in the RPE is a crucial contributor to the development of AMD. Based on our in vivo findings which raised the possibility that IL-17A might have a protective effect, we further clarified whether IL-17A can protect against oxidative stress in RPE cells. To confirm this hypothesis, we examined the viability of human ARPE-19 cells after IL-17A treatment by using an MTT assay. We found that IL-17A did not affect the viability of ARPE-19 cells (Fig. 4A). To determine the appropriate concentration of H_2_O_2_ to test the protective effect of IL-17A on H_2_O_2_-induced cytotoxicity, the viability of ARPE-19 cells was examined after H_2_O_2_ (0–2 mM) exposure for 24 h. We found that the IC_50_ value of H_2_O_2_ after stimulation for 24 h was 300 μM (data not shown). Therefore, we selected this experimental condition for the following experiments. Next, ARPE-19 cells were cotreated with IL-17A and H_2_O_2,_ and then cell viability was analyzed. MTT assays showed that IL-17A did not affect ARPE-19 cell viability during exposure to H_2_O_2_-induced oxidative stress (Fig. 4B). We used the Annexin V/PI apoptosis assay to clarify the effect of IL-17A during exposure to oxidative stress. FACS analysis showed that IL-17A inhibited cell apoptosis during exposure to H_2_O_2_-induced oxidative stress in ARPE-19 cells (Fig. 4C, D).

### IL-17A induced the expression of the antioxidant protein HO-1 during exposure to H_2_O_2_-induced oxidative stress

Previous studies [39, 40] reported the essential role of the Nrf2/Keap1/ARE pathway in redox homeostasis; this pathway leads to the expression of cytoprotective enzymes, such as NAD(P)H: quinone oxidoreductase 1 (NQO1) and heme oxygenase 1 (HO-1), to counteract oxidative stress. The Nrf2 pathway and its downstream antioxidant enzyme HO-1 are crucial for cellular defense against H_2_O_2_-induced oxidative damage [41]. HO-1 upregulation inhibited H_2_O_2_-induced apoptosis in ARPE-19 cells [42]. RT‒qPCR showed that H_2_O_2_ treatment induced the expression of HO-1 and NQO1. H_2_O_2_ combined with IL-17A treatment induced more HO-1 but no NQO1 expression compared with H_2_O_2_ treatment alone in ARPE-19 cells (Fig. 4E, F). Western blotting showed that IL-17A treatment increased HO-1 protein levels during exposure to H_2_O_2_-induced oxidative stress (Fig. 4G, H).

### IL-17A facilitates repair of oxidative stress-induced barrier dysfunction

**Fig. 5:**
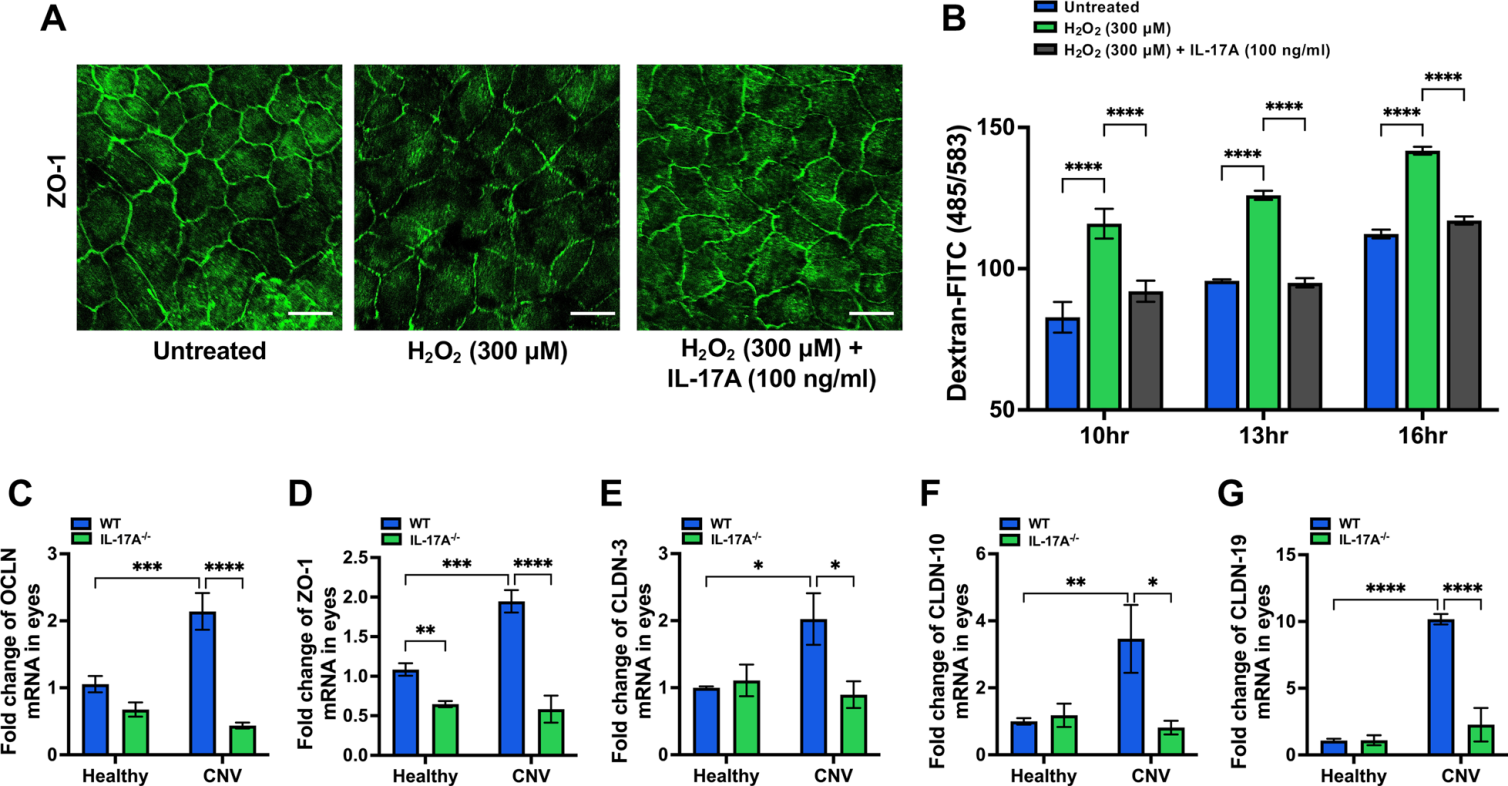
IL-17A facilitated repair of oxidative stress-induced barrier dysfunction. ARPE-19 cells were cultured in DMEM/F12 with 1% FBS for 7 days until they reached the appropriate cell confluence. **A** ARPE-19 cells were cotreated with 300 μM H_2_O_2_ and IL-17A (100 ng/ml) in the Transwell insert for 16 h, and immunofluorescence staining was performed with an anti-ZO-1-specific antibody in ARPE-19 monolayers in vitro. Scale bar = 75 μm. **B** To analyze the permeability function, ARPE-19 cells were cotreated with 300 μM H_2_O_2_ and IL-17A (100 ng/ml) in the Transwell insert for 16 h. FITC-dextran (100 μg/ml) was added after treatment, and the fluorescent content was measured at 485/538 nm excitation/emission wavelengths using a luminometer at 10-16 h. Statistical differences were determined by one-way ANOVA and Tukey’s multiple comparisons test. Data are presented as the mean ± SEM (****P < 0.0001). The data shown are representative of three independent experiments with similar results. **C**–**G** The mRNA expression of ZO-1, OCLN, CLDN-3, CLDN-10, and CLDN-19 in eyes isolated from WT and IL-17A^−/−^ mice (N = 6) at 28 days after laser injury was analyzed using RT‒qPCR with specific primers. Statistical differences were determined by two-way ANOVA and Sidak's multiple comparisons test. Data are presented as the mean ± SEM (*P < 0.05, **P < 0.01, ***P < 0.001, ****P < 0.0001). The data shown are representative of three independent experiments with similar results

The outer blood‒retina barrier (BRB) formed by the tight junctions between RPE cells is critical for maintaining retinal homeostasis. The integrity of the outer BRB prevents choroidal neovascularization from invading the retina [43, 44]. The components of the BRB are tight junction proteins, including zonula occludens 1 (ZO-1), occludin (OCLN), and the claudin (CLDN) family [45]. A previous study [46] indicated that the HO-1-dependent MAPK pathway regulated intestinal barrier disruption. Our in vitro findings showed that IL-17A treatment increased HO-1 protein levels during exposure to H_2_O_2_-induced oxidative stress, suggesting that IL-17A may play a role in maintaining barrier function in the retina. To confirm this hypothesis, we used ARPE-19 cells to establish an in vitro model to evaluate the effect of IL-17A on retina barrier function. Immunofluorescence staining of the tight junction marker ZO-1 showed that the ARPE-19 monolayer successfully formed a tight junction in the untreated control group (Fig. 5A). We observed that H_2_O_2_ treatment destroyed the ARPE-19 monolayer, which suggested that H_2_O_2_ can damage the RPE by disrupting tight junctions. Compared with H_2_O_2_ treatment alone, IL-17A and H_2_O_2_ cotreatment preserved the integrity of the whole monolayer (Fig. 5A). Additionally, we set up a FITC-dextran permeability assay to evaluate oxidative stress-induced barrier dysfunction in ARPE-19 cells. We found that the FITC-dextran concentration in the H_2_O_2_-treated group increased in a time-dependent manner compared to that in the untreated control group. The concentration of FITC-dextran was significantly reduced in the IL-17A and H_2_O_2_ cotreated group compared with the H_2_O_2_-treated group (Fig. 5B). To further verify that IL-17A has a beneficial effect on the restoration of oxidative stress-induced barrier dysfunction, we analyzed tight junction proteins, including ZO-1, OCLN, and the CLDN family, in the eyes of mice with laser-induced CNV. RT‒qPCR showed that the expression of ZO-1, OCLN, CLDN-3, CLDN-10, and CLDN-19 in the eyes was decreased in IL-17A-deficient mice compared with WT mice at 28 days after laser injury (Fig. 5C–G). These data suggested that IL-17A plays a role in maintaining retinal homeostasis and impacts the repair of oxidative stress-induced barrier dysfunction in the laser-induced CNV mouse model.

## Discussion

Oxidative stress is a critical inducer of RPE cell dysregulation in wet AMD; this process causes RPE barrier dysfunction and might result from disruption of intercellular tight junctions. We demonstrated that IL-17A deficiency exacerbated disease severity in a laser-induced CNV mouse model. IL-17A deficiency influenced ocular Treg cells, CD3^+^CD4^+^RORγt^+^ T cells, γδ T cells, macrophages, and neutrophils and affected the expression of ocular proinflammatory molecules. IL-17A inhibited cell apoptosis and induced the expression of the antioxidant protein HO-1 in ARPE-19 cells under oxidative stress. IL-17A has the ability to repair oxidative stress-induced barrier dysfunction in ARPE-19 cells. These findings suggest a protective effect of IL-17A in the laser-induced CNV model.

To confirm the elevated serum IL-17A levels in AMD patients, we established a laser-induced CNV mouse model and found that IL-17A was expressed mainly in CNV lesions. We did not observe a significant change in serum IL-17A levels in mice after laser injury. However, we found that the number of IL-17A-expressing cells in the eyes increased after laser injury. IL-17A deficiency influenced Treg, Th17, and γδ T-cell populations in the eyes but not in the cervical lymph nodes. In addition, IL-17A deficiency decreased neutrophil infiltration and increased macrophage infiltration into the retina. Therefore, the laser-induced CNV mouse model might involve a local inflammatory response and disruption of the ocular microenvironment but not the systemic microenvironment 28 days after laser injury. In addition, we analyzed the dynamic changes in the proportions of IL-17A-expressing immune cells and observed that the number of γδ T cells significantly increased from day 7 to 28 after laser injury; this change occurred in a time-dependent manner. These data suggested that IL-17A-secreting γδ T cells might play a critical role in maintaining ocular homeostasis at later time points after laser-induced injury.

Previous studies [47–49] reported that CNV lesions trigger local ocular inflammation via the infiltration of IL-17-producing γδ T cells, which are presumably recruited to the eye in a manner dependent on complement factor 5a. IL-17 generates a proinflammatory environment in the RPE by affecting barrier function. Gene transfer of a soluble IL-17 receptor prevents retinotoxicity in DKO/rd8 mice [50]. In addition, IL-17A deficiency in mice was reported to inhibit CNV development in the early phase after laser injury [51]. These findings provide evidence for a pathogenic role of IL-17A in AMD. Since AMD is an age-related progressive chronic disease, we speculated that the dynamic change in the laser-induced CNV model needs to be observed for a longer time. We observed no significant change in CNV lesions between WT and IL-17A-deficient mice at 7 days after laser injury. However, unexpectedly, the size of CNV lesions was significantly increased in IL-17A-deficient mice at 14, 21, and 28 days after laser injury. IL-17A deficiency caused a larger area of damage to the retinal structure. These data suggested that IL-17A might play a protective role in the laser-induced CNV mouse model.

Clinical trials targeting IL-17A in rheumatoid arthritis [52] and uveitis [53] have reported disappointing results. A previous study [54] reported that an IL-17A negative feedback loop limited Th17 pathogenicity in experimental autoimmune uveitis (EAU). The authors revealed that IL-17A could bind its receptor and further trigger the Th17 cell-intrinsic autocrine loop, which activates the transcription factor NF-κB and induces IL-24; IL-24 can then further suppress the expression of the Th17-lineage cytokines IL-17F and GM-CSF. A recent study [55] reported that IL-24 suppressed the production of proinflammatory cytokines and chemokines in ocular-infiltrating Th1 and Th17 cells and RPE cells in the EAU mouse model. IL-24 significantly suppressed the expression of RORγt, the master regulator of Th17 cells, and inhibited IL-17A production by Th17 cells. In our in vivo data, we found that IL-17F, GM-CSF, and IL-24 levels were significantly reduced in IL-17A-deficient mice compared with WT mice at 28 days after laser injury. These data indicated that the IL-17A-IL-24 circuit functions through a negative feedback mechanism and is critical for regulating ocular inflammation.

Previous studies [23, 33, 56, 57] have demonstrated that γδ T-cell-produced IL-17 can activate neutrophils and initiate an antibacterial immune response against infections occurring in several tissues. However, it is also important to note that excessive IL-17 production and the resultant neutrophilia can lead to immunopathology, further contributing to the onset or exacerbation of inflammatory diseases. These results support the idea that IL-17A may be a double-edged sword in terms of its effects during the early and late stages of laser-induced CNV in mice. On the other hand, a previous study [58] showed that in the absence of γδ T cells, bleomycin-stimulated mice showed more severe pulmonary interstitial inflammation and a significantly lower level of IL-17 production. The deficiency of IL-17 secretion resulted in a notable delay in epithelial regeneration following bleomycin instillation. Consistently, our study showed that the laser-induced inflammation and epithelial destruction in IL-17A-deficient CNV mice were more severe than those in WT CNV mice. These results implied that the specific tissue environment dictates the effects of γδ T-cell-produced IL-17A, allowing the immune system to respond to different disease-induced injuries. Our study suggested that the protective function of IL-17A produced by γδ T cells in the eyes involves more than just regulating ocular inflammation; it also involves antioxidant activities that promote the restoration of epithelial integrity.

A previous study [59] reported that macrophages activated under conditions of oxidative stress could enhance the inflammatory response but impair the phagocytic response, leading to ineffective clearance of apoptotic cells. Therefore, we speculated that IL-17A deficiency might cause a high level of oxidative stress and further direct macrophage activation to promote a robust inflammatory response that causes larger CNV lesions. Whether IL-17A-mediated oxidative stress promotes macrophage infiltration/activation and causes larger CNV lesions awaits further investigation. Future studies are needed to clarify these mechanisms by evaluating the effect of ROS inhibitor treatment in IL-17A-deficient mice with laser-induced CNV.

IL-10 was upregulated in ocular macrophages of aged mice after laser injury. In addition, IL-10 regulates macrophage function and promotes angiogenesis [19]. IL-6 was shown to be associated with the volume of macular edema in patients with CNV [18]. In our in vivo data, IL-10 expression was upregulated in the eyes of IL-17A-deficient mice 7 days after laser injury. IL-6 expression was increased in the eyes and serum of IL-17A-deficient mice 28 days after laser injury. We speculated that IL-10 might influence macrophage clearance of apoptotic cells in IL-17A-deficient mice at 7 days and further increase macrophage infiltration into the retina at 28 days after laser injury. Therefore, IL-10 may directly cause CNV lesion expansion at 14, 21, and 28 days.

Th17 cells, ILC3s, and γδ T cells mainly produce IL-17A. Neutrophils and macrophages also secrete IL-17A. In addition, a previous study [60] reported that intestinal Paneth cells produce IL-17A in an IL-23-independent fashion, which is one of the causes of systemic inflammatory response syndrome. IL-17A and IL-17F are expressed in colonic [61] and lung epithelial cells [62]. Our study also showed that nonimmune cells can secrete IL-17A in the retina. However, the role of IL-17A-expressing nonimmune cells in the development of AMD requires further investigation.

IL-17A acts as a pathogenic cytokine in a variety of inflammatory diseases, including multiple sclerosis [26, 27], inflammatory bowel disease [29, 30], and uveitis [63–66]. A previous study [29] reported that IL-17A-producing resident γδ T cells are essential for maintaining and protecting the epithelial barriers in the intestinal mucosa. The data from that study demonstrated one possible mechanism by which IL-17A signaling through Act-1 maintains OCLN localization to support barrier function during DSS-induced injury. Within the RPE, CLDN and OCLN traverse the plasma membrane, where ZO proteins anchor them. This anchoring enables their binding with signaling molecules and the actin cytoskeleton, thereby promoting the formation and stabilization of tight junctions [45]. Although the CLDN family includes at least 27 members, their expression patterns vary in different organs and tissues, contributing to the tissue-specific characteristics of tight junctions [67]. CLDN-19 is predominantly expressed in the human RPE and is essential for maintaining RPE tight junction formation and barrier function [68]. A previous study showed that patients harboring mutations in the CLDN-19 gene in the RPE suffer severe ocular impairment [69]. In addition, there is detectable, albeit lower, expression of CLDN-3 and CLDN-10 in RPE cells [70]. Therefore, we examined the expression of OCLN, ZO-1, CLDN-3, CLDN-10, and CLDN-19 in our laser-induced CNV model, and we found that these tight junction genes were expressed at lower levels in IL-17A-deficient mice than in WT mice. The laser injured the RPE and Bruch’s membrane and caused RPE barrier and membrane rupture [71], which may explain why the levels of tight junction genes were decreased at 28 days after laser injury.

Previous studies reported that IL-17A promoted VEGF production in corneal neovascularization [72], osteoarthritis [73], and rheumatoid arthritis [74, 75]. In our in vivo data, IL-17A deficiency did not influence VEGF expression in the laser-induced CNV mouse model. The retina resides in an environment that is primed for the generation of ROS and resultant oxidative damage. Oxidative stress contributes to the production of proangiogenic factors in the retina [76]. Previous studies have reported that many chemical compounds can induce the production of antioxidant proteins, including HO-1 and NQO-1, through the Nrf2 pathway to inhibit ROS production and further protect against oxidative stress [77–80]. According to our in vitro data, IL-17A-induced HO-1 expression might occur via the Nrf2 pathway. Therefore, we speculated that IL-17A might play a critical role in activating the oxidative stress-associated pathway without directly influencing the VEGF-associated signaling pathway in the laser-induced CNV mouse model. In addition, a previous study showed that VEGF expression peaked 7 days after laser injury [81]. Our data showed that VEGF expression could be detected at 28 days after laser injury. However, we cannot exclude the possibility that the use of different observation time points led us to miss a difference in VEGF expression between WT and IL-17A-deficient mice.

Intravitreal injections of anti-VEGF agents have become a first-line treatment for wet AMD. However, some patients still have persistent fluid or recurrent exudation, which is called refractory neovascular AMD [82]. A previous study also reported many obvious markers of oxidative damage in AMD, and antioxidant therapy is a potential treatment for AMD [83]. However, whether refractory neovascular AMD patients have high levels of oxidative stress in the retina is still unknown. According to our in vitro data, IL-17A treatment induced a high level of antioxidant proteins in APRE-19 cells. Future studies are needed to clarify the possible effect of combination treatment with IL-17A and anti-VEGF in animal models.

In summary, our findings demonstrate a possible effect of IL-17A-producing γδ T cells on the progression of laser-induced CNV in mice. IL-17A can exert a protective effect against laser-induced CNV injury. The underlying mechanism is that IL-17A induces the expression of the antioxidant protein HO-1, which might inhibit RPE cell apoptosis and prevent redundant macrophage infiltration into the retina and the subsequent generation of an excessive immune response. IL-17A repaired oxidative stress-induced barrier dysfunction. Our findings provide insight into the protective mechanisms of IL-17A in the ocular microenvironment, which may provide a new direction for understanding wet AMD.

### Supplementary Information

**Additional file 1: Figure S1.** Expression of VEGFA in the laser-induced CNV mouse model. **A** Immunofluorescence staining for VEGFA expression in ocular tissue in WT and IL-17A^−/−^ mice 28 days after laser injury (N = 4). Magnification: 200X. **B**, **C** The ocular tissues from WT and IL-17A^−/−^ mice 28 days after laser injury (N = 3) were collected and analyzed using immunoblotting with specific antibodies against VEGFA. GAPDH was used as an internal control. **D** The mRNA expression of VEGFA in eyes isolated from WT and IL-17A^−/−^ mice (N = 6) 28 days after laser injury was analyzed using RT‒qPCR with specific primers. Statistical differences were determined by two-way ANOVA and Sidak’s multiple comparisons test. Data are presented as the mean ± SEM (***P < 0.001, ****P < 0.0001). **Figure S2.** Analysis of CD3^+^CD4^+^RORγt^+^ T cells, γδ T cells, and Treg cells in cervical lymph nodes of mice in a laser-induced CNV mouse model. **A**–**C** Representative flow cytometry data of CD3^+^CD4^+^RORγt^+^ T cells, γδ T cells, and Treg cells in cervical lymph nodes of WT and IL-17A^−/−^ mice at 28 days after laser injury. N = 5 mice per group. Data are presented as the mean ± SEM. The data shown are representative of three independent experiments with similar results. **Figure S3.** Gating strategy for flow cytometry analysis for Fig. 1. In this sample gating, cells were gated in an SSC-A and FSC-A dot plot to select live cells and then gated in an FSC-H and FSC-A dot plot to eliminate doublets. The singlet gate was then gated on the CD45^+^ population. These were then further gated for the subsets of interest, namely, CD3^+^CD4^+^ T cells, CD3^+^CD4^+^RORγt^+^ T cells, Treg cells (CD3^+^CD4^+^Foxp3^+^), γδ T cells (CD3^+^TCRγδ^+^), macrophages (CD11b^+^F4/80^+^), MDSCs (CD11b^+^Ly6G^−^Ly6C^hi^), and neutrophils (CD11b^+^Ly6G^+^Ly6C^+^). Data were analyzed using FlowJo software, and population frequencies were expressed as percentages of the CD45^+^ parent population. **Figure S4.** Gating strategy for flow cytometry analysis for Fig. 2. In this sample gating, cells were gated in an SSC-A and FSC-A dot plot to select live cells and then gated in an FSC-H and FSC-A dot plot to eliminate doublets. The singlet gate was then gated on the IL-17A^+^ population. These were then further gated for the subsets of interest, namely, IL-17A^+^ immune cells (IL-17A^+^CD45^+^), IL-17A^+^ nonimmune cells (IL-17A^+^CD45^−^), CD3^+^IL-17A^+^ T cells, CD3^+^CD4^+^IL-17A^+^ T cells, CD3^+^CD8^+^IL-17A^+^ T cells, γδ T cells (CD3^+^TCRγδ^+^IL-17A^+^), Th17 cells (CD3^+^CD4^+^RORγt^+^IL-17A^+^), and Treg cells (CD3^+^CD4^+^Foxp3^+^IL-17A^+^). Data were analyzed using FlowJo software, and population frequencies were expressed as percentages of the IL-17A^+^ parent population. **Figure S5.** Dynamic changes in systemic cytokine levels in a laser-induced CNV mouse model. Serum samples were collected from healthy mice and WT and IL-17A^−/−^ mice with laser-induced CNV (N = 5) at 7 days, 14 days, 21 days, and 28 days after laser injury. **A**–**I** The cytokine levels of IL-17F, IL-17A, IL-22, IL-23, TNF-α, IFN-γ, IL-5, IL-13, and IL-6 were measured by LEGENDplex™. Statistical differences were determined by two-way ANOVA and Sidak's multiple comparisons test. Data are presented as the mean ± SEM (*P < 0.05, **P < 0.01, ***P < 0.001). The data shown are representative of two independent experiments with similar results. **Table S1.** List of primers sequences used in this study.

## Data Availability

All data generated or analyzed during this study are included in this published article.

## Abbreviations

AMD: Age-related macular degeneration
CNV: Choroidal neovascularization
IL-17A: Interleukin-17A
WT: Wild-type
FACS: Fluorescence-activated cell sorting
VEGF: Vascular endothelial growth factor
RPE: Retinal pigment epithelium
Th17 cells: T helper 17 cells
ILC3: Type three innate lymphoid cells
γδ T cells: Gamma delta T cells
IBD: Inflammatory bowel diseases
DR: Diabetic retinopathy
EAU: Experimental autoimmune uveitis
ELISA: Enzyme-linked immunosorbent assay
RT‒PCR: Reverse transcription polymerase chain reaction
RT‒qPCR: Real time quantitative polymerase chain reaction
PBS: Phosphate-buffered saline
HRP: Horseradish peroxidase
TNF-α: Tumor necrosis factor-α
IHC: Immunohistochemistry
IF: Immunofluorescence
ZO-1: Zonula occludens-1
OCLN: Occludin
NQO-1: NAD(P)H quinone dehydrogenase-1
HO-1: Heme oxygenase-1
