# Supplementary material for: Protective role of IL-17-producing γδ T cells in a laser-induced choroidal neovascularization mouse model
Source: J Neuroinflammation. 2023 Nov 25;20:279. doi: 10.1186/s12974-023-02952-1 (PMC10676594; doi:10.1186/s12974-023-02952-1)
Supplement: Supplementary file 1 — Additional file 1: Figure S1. Expression of VEGFA in the laser-induced CNV mouse model. A Immunofluorescence staining for VEGFA expression in ocular tissue in WT and IL-17A−/− mice 28 days after laser injury (N = 4). Magnification: 200X. B, C The ocular tissues from WT and IL-17A−/− mice 28 days after laser injury (N = 3) were collected and analyzed using immunoblotting with specific antibodies against VEGFA. GAPDH was used as an internal control. D The mRNA expression of VEGFA in eyes isolated from WT and IL-17A−/− mice (N = 6) 28 days after laser injury was analyzed using RT‒qPCR with specific primers. Statistical differences were determined by two-way ANOVA and Sidak’s multiple comparisons test. Data are presented as the mean ± SEM (***P < 0.001, ****P < 0.0001). Figure S2. Analysis of CD3+CD4+RORγt+ T cells, γδ T cells, and Treg cells in cervical lymph nodes of mice in a laser-induced CNV mouse model. A–C Representative flow cytometry data of CD3+CD4+RORγt+ T cells, γδ T cells, and Treg cells in cervical lymph nodes of WT and IL-17A−/− mice at 28 days after laser injury. N = 5 mice per group. Data are presented as the mean ± SEM. The data shown are representative of three independent experiments with similar results. Figure S3. Gating strategy for flow cytometry analysis for Fig. 1. In this sample gating, cells were gated in an SSC-A and FSC-A dot plot to select live cells and then gated in an FSC-H and FSC-A dot plot to eliminate doublets. The singlet gate was then gated on the CD45+ population. These were then further gated for the subsets of interest, namely, CD3+CD4+ T cells, CD3+CD4+RORγt+ T cells, Treg cells (CD3+CD4+Foxp3+), γδ T cells (CD3+TCRγδ+), macrophages (CD11b+F4/80+), MDSCs (CD11b+Ly6G−Ly6Chi), and neutrophils (CD11b+Ly6G+Ly6C+). Data were analyzed using FlowJo software, and population frequencies were expressed as percentages of the CD45+ parent population. Figure S4. Gating strategy for flow cytometry analysis for Fig. 2. In this sample gating, [file 12974_2023_2952_MOESM1_ESM.docx]

**Additional file**

**
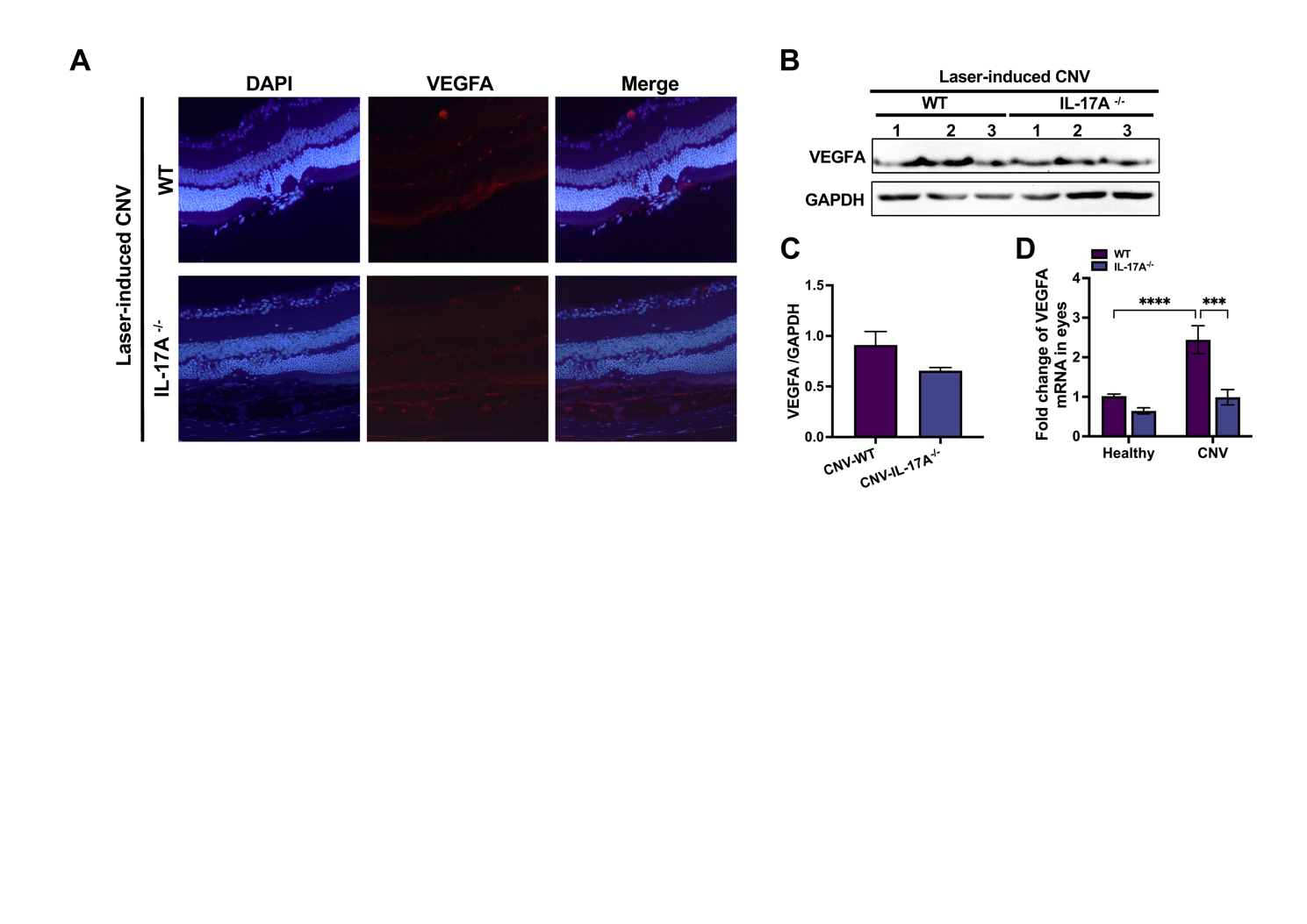
**

**Fig. S1. Expression of VEGFA in the laser-induced CNV mouse model.**

(**A**) Immunofluorescence staining for VEGFA expression in ocular tissue in WT and IL-17A^-/-^ mice 28 days after laser injury (N = 4). Magnification: 200X. (**B**-**C**) The ocular tissues from WT and IL-17A^-/-^ mice 28 days after laser injury (N = 3) were collected and analyzed using immunoblotting with specific antibodies against VEGFA. GAPDH was used as an internal control. (**D**) The mRNA expression of VEGFA in eyes isolated from WT and IL-17A^-/-^ mice (N = 6) 28 days after laser injury was analyzed using RT‒qPCR with specific primers. Statistical differences were determined by two-way ANOVA and Sidak's multiple comparisons test. Data are presented as the mean ± SEM (***P<0.001, ****P<0.0001).


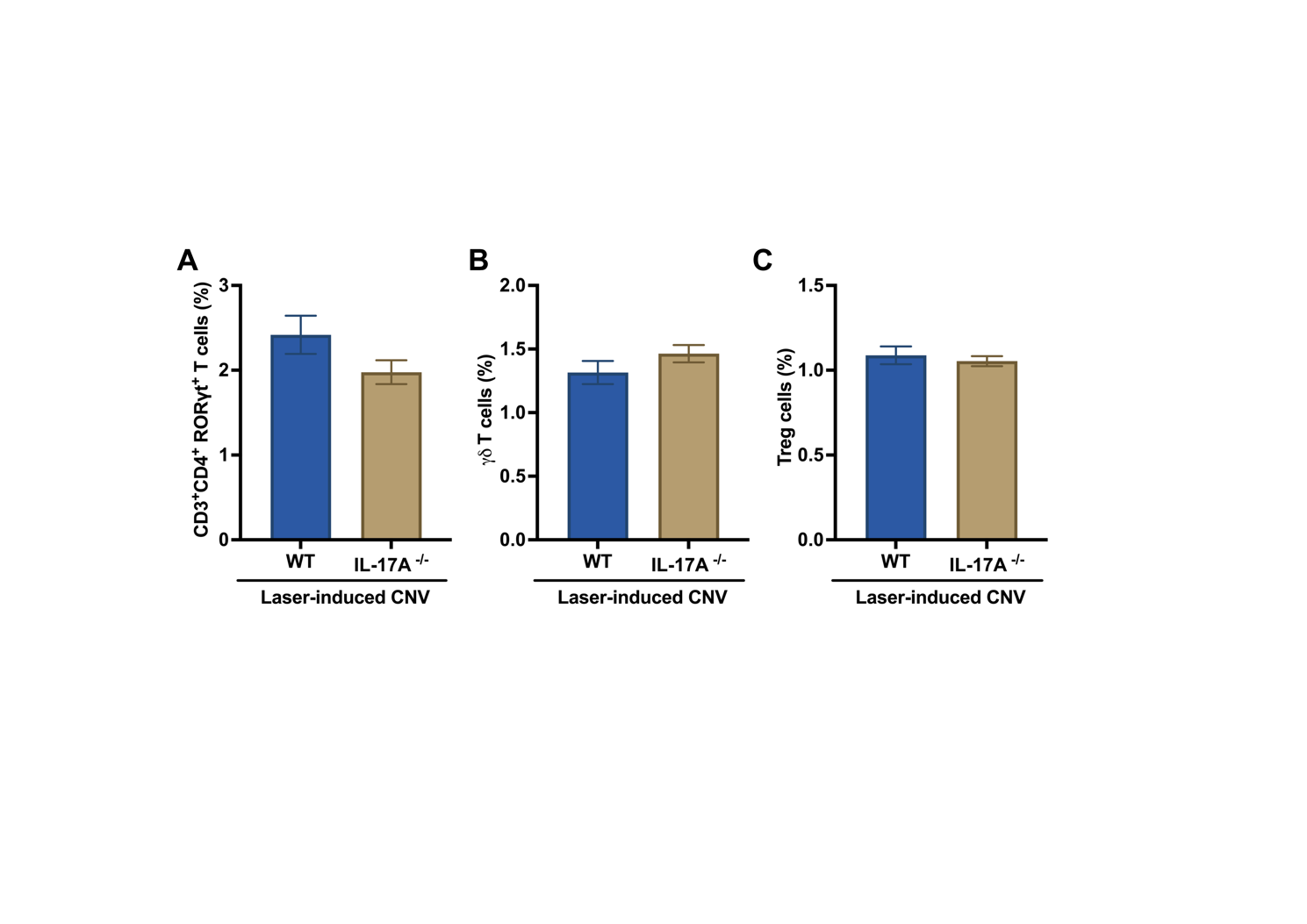


**Fig. S2. Analysis of CD3^+^CD4^+^RORγt^+^ T** **cells, γδ T cells, and Treg cells in cervical lymph nodes of mice in a laser-induced CNV mouse model.**

(**A**-**C**) Representative flow cytometry data of CD3^+^CD4^+^RORγt^+^ T cells, γδ T cells, and Treg cells in cervical lymph nodes of WT and IL-17A^-/-^ mice at 28 days after laser injury. N = 5 mice per group. Data are presented as the mean ± SEM. The data shown are representative of three independent experiments with similar results.

**
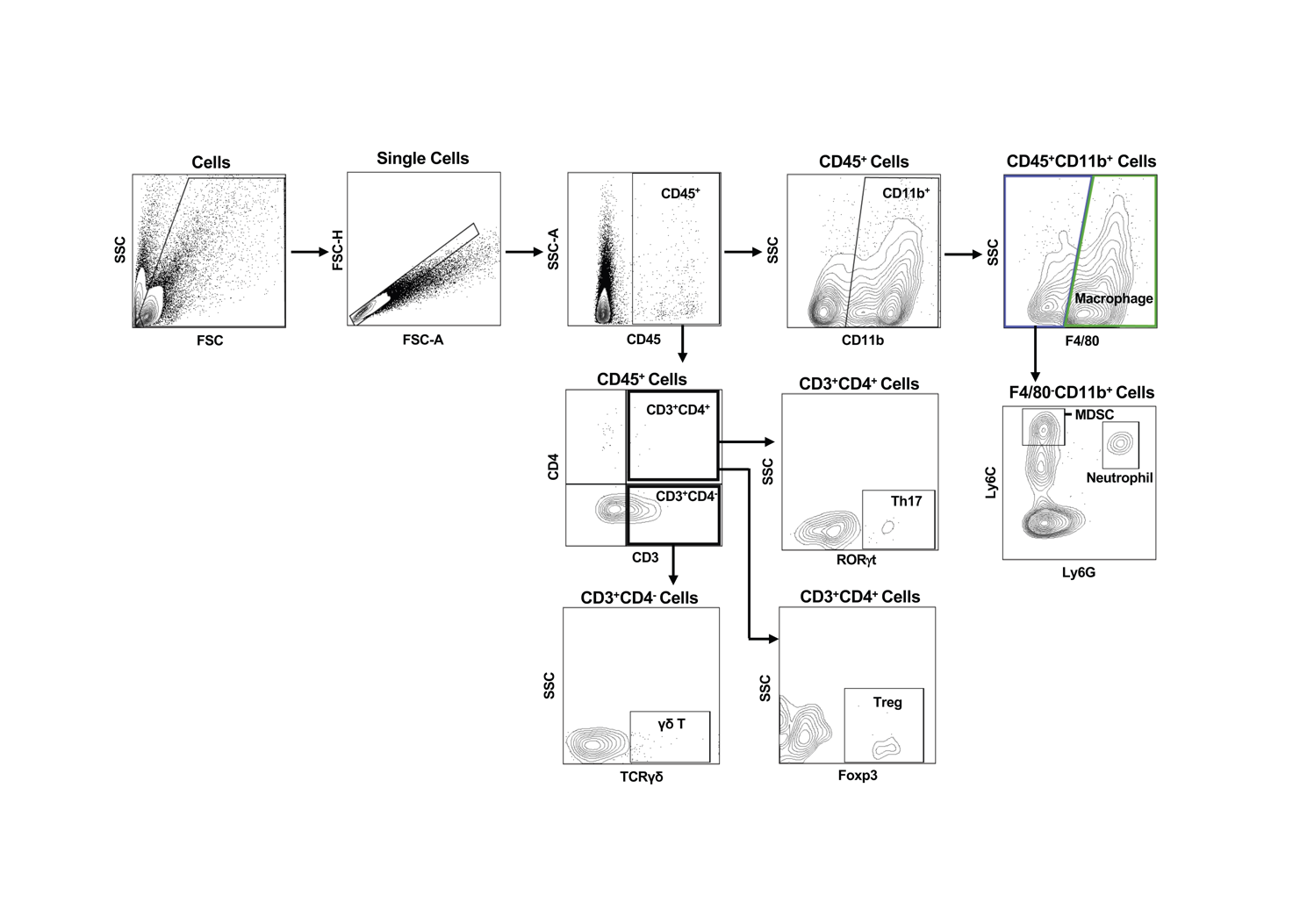
**

**Fig. S3. Gating strategy for flow cytometry analysis for Fig. 1**.

In this sample gating, cells were gated in an SSC-A and FSC-A dot plot to select live cells and then gated in an FSC-H and FSC-A dot plot to eliminate doublets. The singlet gate was then gated on the CD45^+^ population. These were then further gated for the subsets of interest, namely, CD3^+^CD4^+^ T cells, CD3^+^CD4^+^RORγt^+^ T cells, Treg cells (CD3^+^CD4^+^Foxp3^+^), γδ T cells (CD3^+^TCRγδ^+^), macrophages (CD11b^+^F4/80^+^), MDSCs (CD11b^+^Ly6G^-^Ly6C^hi^), and neutrophils (CD11b^+^Ly6G^+^Ly6C^+^). Data were analyzed using FlowJo software, and population frequencies were expressed as percentages of the CD45^+^ parent population.


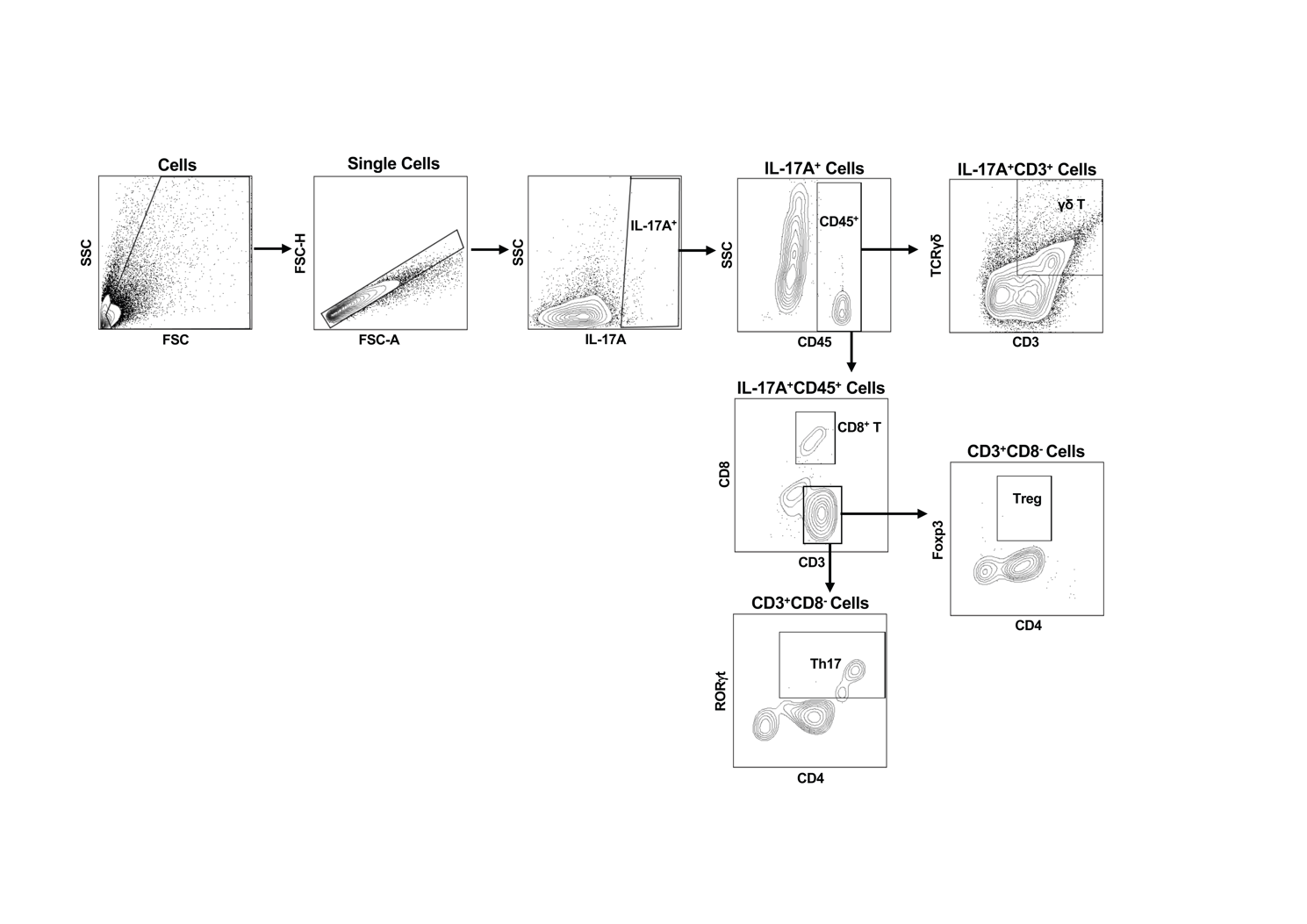


**Fig. S4. Gating strategy for flow cytometry analysis for Fig. 2.**

In this sample gating, cells were gated in an SSC-A and FSC-A dot plot to select live cells and then gated in an FSC-H and FSC-A dot plot to eliminate doublets. The singlet gate was then gated on the IL-17A^+^ population. These were then further gated for the subsets of interest, namely, IL-17A^+^ immune cells (IL-17A^+^CD45^+^), IL-17A^+^ nonimmune cells (IL-17A^+^CD45^-^), CD3^+^IL-17A^+^ T cells, CD3^+^CD4^+^IL-17A^+^ T cells, CD3^+^CD8^+^IL-17A^+^ T cells, γδ T cells (CD3^+^TCRγδ^+^IL-17A^+^), Th17 cells (CD3^+^CD4^+^RORγt^+^IL-17A^+^), and Treg cells (CD3^+^CD4^+^Foxp3^+^IL-17A^+^). Data were analyzed using FlowJo software, and population frequencies were expressed as percentages of the IL-17A^+^ parent population.


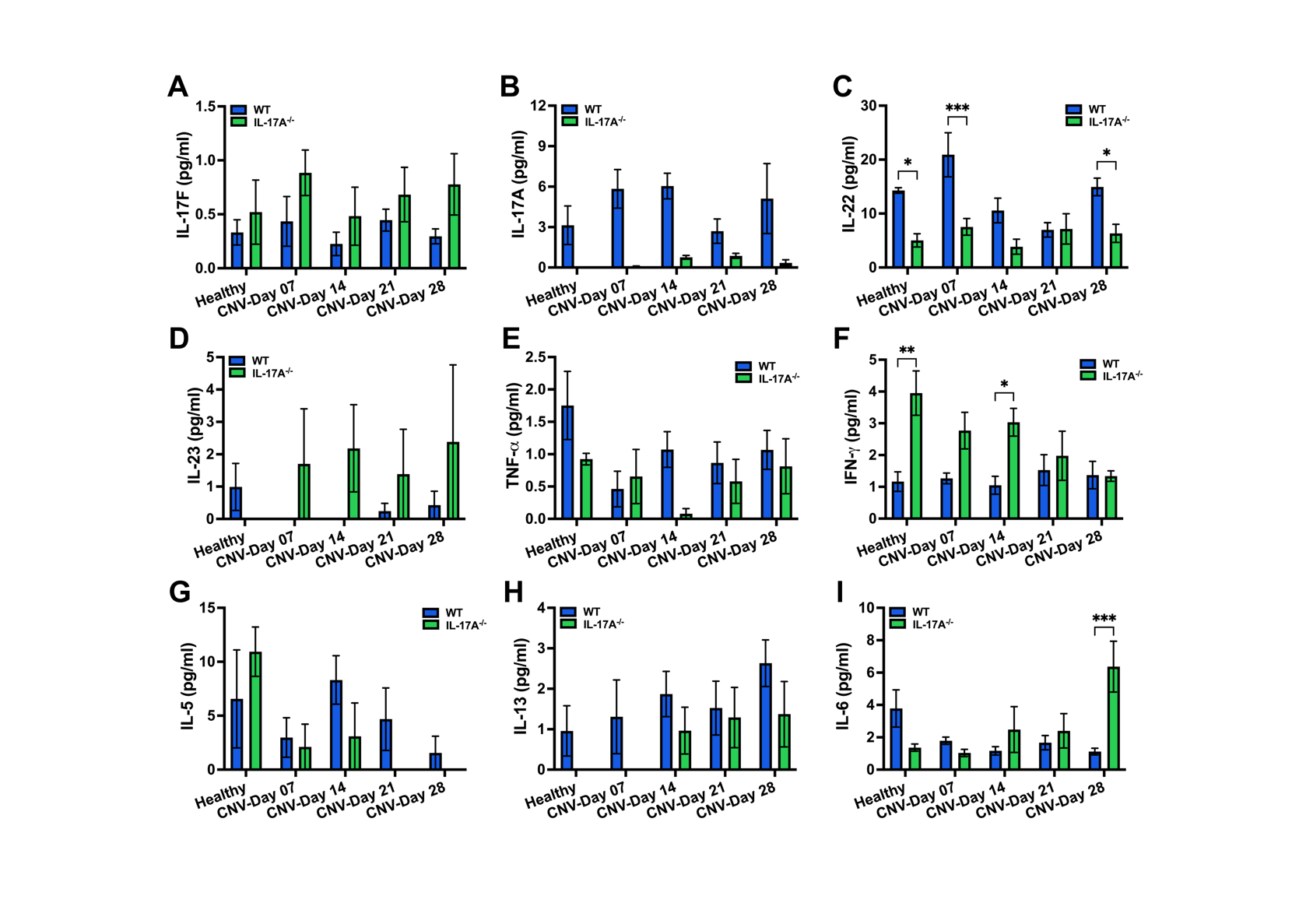


**Fig. S5. Dynamic changes in systemic cytokine levels in a laser-induced CNV mouse model.**

Serum samples were collected from healthy mice and WT and IL-17A^-/-^ mice with laser-induced CNV (N = 5) at 7 days, 14 days, 21 days, and 28 days after laser injury. (**A**-**I**) The cytokine levels of IL-17F, IL-17A, IL-22, IL-23, TNF-α, IFN-γ, IL-5, IL-13, and IL-6 were measured by LEGENDplex™. Statistical differences were determined by two-way ANOVA and Sidak's multiple comparisons test. Data are presented as the mean ± SEM (*P<0.05, **P<0.01, ***P<0.001). The data shown are representative of two independent experiments with similar results.

## Table S1. List of primers sequences used in this study

| **Mouse** | | |
| --- | --- | --- |
| **Gene** | **Forward primer (5’−3’)** | **Reverse primer (5’−3’)** |
| **β**-**actin** | GAAGTGTGACGTTGACATCC | GTACTCCTGCTTGCTGATC |
| **IL-1β** | CCTGTGTAATGAAAGACGGC | CTGCTTGTGAGGTGCTGATG |
| **TNF-α** | GCCTCTTCTCATTCCTGCTTG | CTGATGAGAGGGAGGCCATT |
| **IL-6** | GATGGATGCTACCAAACTGG | GAAATTGGGGTAGGAAGGAC |
| **IL-10** | CCGACTCCTTAATGCAGGAC | GTAGACACCTTGGTCTTGGAG |
| **OCLN** | CACCCCCATCTGACTATGCG | TTTCCTGCTTTCCCCTTCGT |
| **ZO-1** | CCAAATGCGGTTGATCGTCTT | GGCACCGTACCAACCATCA |
| **MCP-1** | CTGGAGAGCTACAAGAGGAT | CTTCACATTCAAAGGTGCTG |
| **VEGFA** | GTTCCAACCAGAAGTTTGGGG | TTCTTCCACCACCGTGTCTTC |
| **TGF-β** | GGATACCAACTATTGCTTCAG | ACCTTGCTGTACTGTGTGTCC |
| **CLDN-3** | GATGGGAGCTGGGTTGTACG | CTGGTAGTGGTGACGGTACG |
| **CLDN-10** | ACACATACAACGGACCCACG | GAGAGCCTTGGAAGGACTATTG |
| **CLDN-19** | CCCAGCACTCCTGTCAATGC | AGTTGAGGGTCCAGAGCGATA |
| **IL-24** | GATGACATCACAAGCATCCG | ACTTCAGCAGGCTGTGGG |
| **IL-17F** | CTGGATTCTACTGCATGACCCG | GGCAAGTCCCAACATCAACAG |
| **GM-CSF** | TCACCCATCACTGTCACCCG | GTCTGGTAGTAGCTGGCTGTC |
| **Human** | | |
| **Gene** | **Forward primer (5’−3’)** | **Reverse primer (5’−3’)** |
| **GAPDH** | CAGCGACACCCACTCCTC | TGAGGTCCACCACCCTGT |
| **ZO-1** | CAAAGCCAGCGCATTCTCA | ACACTGAATTACCTTCACCATGT |
| **OCLN** | AGGAGGACTGGATCAGGGAAT | TGCAGATCCCTTCACTTGCTT |
| **NQO-1** | CCATGTACTCTCTGCAAGGGAT | CCTGCCTGGAAGTTTAGGTCA |
| **HO-1** | GGCCAGCAACAAAGTGCAAG | GGTAAGGAAGCCAGCCAAGA |
